# Reinforcement learning-guided sample selection for improved identification of phage receptor binding proteins

**DOI:** 10.1093/bib/bbaf631.045

**Published:** 2025-12-12

**Authors:** Xiling Luo, Le Ou-Yang, Yang Shen, Yanni Sun, Jiayu Shang

**Affiliations:** The College of Electronics and Information Engineering, Shenzhen University, 518060, Guangdong, China; SMBU-MSU-BIT Joint Laboratory on Bioinformatics and Engineering Biology, Shenzhen MSU-BIT University, 518172, Guangdong, China; Department of Electrical Engineering, City University of Hong Kong, Hong Kong (SAR), China; Department of Electrical Engineering, City University of Hong Kong, Hong Kong (SAR), China; Department of Information Engineering, Chinese University of Hong Kong, Hong Kong (SAR), China

## Abstract

Receptor binding proteins (RBPs) are critical for bacteriophage infection, mediating the initial recognition and attachment to specific bacterial surface receptors, thereby determining host range and infection specificity. Despite their functional importance, RBPs exhibit high sequence diversity, vary significantly in length (200–2000+ amino acids), and remain poorly annotated in current databases. This low conservation limits the effectiveness of homology-based search methods, which often fail to detect distantly related RBPs. Deep learning models offer a more flexible alternative by learning complex sequence patterns, but face challenges in training due to extreme class imbalance—RBPs are vastly outnumbered by non-RBP phage proteins, leading to inefficient learning and biased predictions. To address this, we propose a deep learning framework enhanced with a reinforcement learning-based sample selection strategy that dynamically prioritizes the most informative negative samples during training. Evaluated on a curated phage protein dataset, our method achieves a precision of 0.9455 and an F1-score of 0.7309, outperforming existing homology-based and deep learning approaches. Our results demonstrate that integrating strategic sample selection significantly improves RBP detection in diverse phage genomes.

